# Perceived burden of care and reported coping strategies and needs for family caregivers of people with mental disorders in Zimbabwe

**DOI:** 10.4102/ajod.v5i1.209

**Published:** 2016-08-24

**Authors:** Bazondlile D. Marimbe, Frances Cowan, Lazarus Kajawu, Florence Muchirahondo, Crick Lund

**Affiliations:** 1 Department of Psychiatry, College of Health Sciences, University of Zimbabwe, Zimbabwe; 2 Research Department of Infection and Population Health, University College London, United Kingdom; 3 Centre for Sexual Health and HIV/AIDS Research: Zimbabwe, Zimbabwe; 4 Alan J Flisher Centre for Public Mental Health, Department of Psychiatry and Mental Health, University of Cape Town, South Africa

## Abstract

**Background:**

Mental health service resources are inadequate in low-income countries, and families are frequently expected to provide care for their relative with a mental disorder. However, research on the consequences of caregiving has been limited in low-income countries, including Zimbabwe.

**Objective:**

The study explored the perceived impact of mental illness, reported coping strategies and reported needs of the family members of persons diagnosed with bipolar affective disorder or schizophrenia attending a psychiatric hospital in Harare, Zimbabwe.

**Methods:**

A purposive sample of 31 family members participated in in-depth interviews and focus group discussions using standardised study guides. Participants were also screened for common mental disorders (CMDs) using the 14-item Shona Symptom Questionnaire. Qualitative data were analysed thematically using NVivo 8 qualitative data analysis software. Statistical Package for Social Sciences (SPSS version 16) was used for descriptive quantitative data analysis.

**Results:**

Caregivers experienced physical, psychological, emotional, social and financial burdens associated with caregiving. They used both emotion-focused and problem-focused coping strategies, depending on the ill family members’ behaviours. Seeking spiritual assistance emerged as their most common way of coping. Twenty-one (68%) of the caregivers were at risk of CMDs (including three participants who were suicidal) and were referred to a psychiatrist for further management. Caregivers required support from healthcare professionals to help them cope better.

**Conclusion:**

Caregivers of patients attending psychiatry hospitals in Zimbabwe carry a substantial and frequently unrecognised burden of caring for a family member with a mental disorder. Better support is needed from health professionals and social services to help them cope better. Further research is required to quantitatively measure caregiver burden and evaluate potential interventions in Zimbabwe.

## Introduction

### Problem statement

Caring for a family member with a mental disorder places an enormous burden on family caregivers and has been shown to have a significant impact on the family’s quality of life (Hsiao *et al*. 2006; Saunders 2003). However, the extent of this burden is often difficult to assess and, partly as a result of these measurement difficulties, is frequently ignored (WHO 2011a).

Mental health service resources are inadequate in low- to middle-income countries (LMICs), in contrast with high-income countries (HICs), where community-based resources such as halfway homes and day-care centres are relatively more available (WHO 2011b). Zimbabwe follows the trend in many LMICs, with major deficits in mental health services (Ministry of Health and Child Welfare 2005; Osaka *et al*. 2010).

### Trends

Whether due to the lack of mental health service resources in LMICs or the history of deinstitutionalisation in many HICs, the burden of care for people with severe mental disorders frequently falls on family members and the communities in which they live (Hsiao *et al*. 2006; Saunders 2003; Yip 2004). Families worldwide are inadequately supported to provide care and support for their relatives with a mental disorder and struggle to look after them (Doornbos 2002; Leung, Au & Lee 2010; Magliano *et al*. 2005; Yip 2004).

In addition to the lack of resources, stigma forms an important part of the experience of people living with mental illness and their family caregivers, including in sub-Saharan Africa. Seventy-five per cent of family members of individuals with schizophrenia reported being stigmatised in Ethiopia, with over one-third (37%) wishing to hide a member with mental disorder (Shibre *et al*. 2003). Stigma has often resulted in strained relationships within the families themselves as well as affecting relationships with neighbours and the society at large (Struening, Perlick & Link 2001; Yang 2007) and this difficulty has been attributed to misunderstanding of mental illness aetiology (Shibre *et al*. 2001). Stigma is linked with depressive symptoms among the family caregivers of individuals with a mental disorder (Perlick *et al*. 2001; Phelan, Bromet & Link 1998). A review by Steel *et al*. (2010) in the USA that sought to assess the psychiatric symptoms in caregivers of persons with bipolar affective disorder showed that 46% of them were depressed, and 32.4% reported using health services themselves.

Coping refers to cognitive and behavioural efforts aimed at managing a troubled person–environmental relationship (Lazarus & Folkman 1985). This includes any response to external life strains that serves to prevent, avoid or control emotional distress and keep an individual away from damage from life strains (Lazarus & Folkman 1985). Coping is related to both the state of one’s inner emotional life and life strains; it depends on subjective well-being, social functioning and somatic health, as well as the relevance placed on these by an individual at any given time (Lazarus & Folkman 1985). Coping is better explained using the transactional model of stress and coping, which emphasises the appraisal to evaluate harm, threats and challenges, resulting in the process of coping with stressful events (Lazarus & Folkman 1984). Coping strategies are summarised under two broad patterns of coping, namely problem-focused and emotion-focused coping mechanisms. Problem-focused coping aims at tackling a problem directly by changing an aspect of a situation, whereas emotion-focused coping attempts either to change the way the stressful environment is viewed or to change the personal meaning of the situation, resulting in separation from the event, escape-avoidance or seeking social approval (Lazarus & Folkman 1984). Both emotion-focused and problem-focused coping strategies are used by caregivers of family members with a mental disorder, with the strategy being determined by demographic attributes of the ill members and caregivers (Magliano *et al*. 1999).

Coping strategies can also be classified as being either positive or negative. Positive thinking and the utilisation of appropriate social supports such as family, friends and the church are some of the aspects of positive coping strategies (Perlick *et al*. 2004), whilst use of avoidance behaviours, negative thinking and substance abuse are some of the examples of negative coping strategies (Menha 2005).

In addition to the above coping strategies, caregivers of family members with either bipolar affective disorder or schizophrenia have been found to require emotional support, psychoeducation and information about the illness for them to effectively provide care and support to their mentally ill relatives (Chakrabarti & Gill 2002). Support groups and counselling have also been found to be helpful in reducing caregiver burden (Mittelman *et al*. 2006).

Although there is burgeoning literature on the impact of mental illness on families in HICs, as indicated by Aschbrenner, Greenberg and Seltzer (2009) and Moller *et al*. (2009), there is still a paucity of published data from low-income countries such as Zimbabwe. Studies have been limited to describing the patient’s illness, such as depression (Abas & Broadhead 1997).

### Aim of the study

We therefore set out to assess the impact of caring for a family member with a mental disorder, as well as the coping strategies and needs among Zimbabwean families of people with mental disorders.

#### Contribution to field

Such data would contribute towards designing culturally sensitive interventions to improve the well-being of caregivers and hence mentally ill people in Zimbabwe.

## Methods and design

### Setting

The study location was one of the two major referral psychiatry hospitals in Harare, the capital city of Zimbabwe. This unit caters for over 200 outpatients per week and has an admission capacity of 36 beds for adults. Service users are referred to this hospital from primary healthcare facilities, regional hospitals and surrounding rural areas. It caters for half of the population of Harare, which is 2 123 132 (Population Census Office 2012).

### Study design

The study used mixed methods, combining both qualitative and quantitative techniques to allow for triangulation of the data, thereby increasing the validity of the findings. An exploratory qualitative approach was taken in an attempt to understand caregiver experiences of caring for a relative with a mental disorder, their coping mechanisms and needs. We adopted this approach as it allows for an opportunity to ‘see through the eyes’ of our participants, a viewpoint that has also been supported by Hedegaard Hakkarainen and Engestrom (1984). Participants were also screened for common mental disorders (CMDs) using the 14-item Shona Symptom Questionnaire (SSQ 14) (Patel *et al*. 1997). The quantitative data helped to ascertain the magnitude of the psychological morbidity among caregivers, which would have not otherwise come to light.

### Recruitment procedure

Participants were purposively selected. They were adult family members who were the primary caregivers of persons diagnosed with schizophrenia or bipolar affective disorder and had accompanied their relatives for either review or admission. All provided written informed consent. We sampled for maximum variation, sampling for diversity across socio-demographic factors and relationship to the patient (sibling, parent, spouse, aunt and children). To recruit participants who met the inclusion criteria, psychiatric nurses working in the unit were asked to identify family members who were waiting in the outpatient clinic with their relative (with a diagnosis of either schizophrenia or bipolar affective disorder) and to refer them to the researcher. All participants were informed about the study and were recruited in line with good clinical practice and asked to sign informed consent. Verbal consent was obtained from the family member with schizophrenia or bipolar affective disorder.

### Instruments and procedure

A structured in-depth interview guide was developed in Shona and used during the individual in-depth interviews with follow-up probes. The questions were open-ended and explored the participants’ experiences of having a relative with a mental disorder; how the illness had affected the family in terms of finances, socialising and their general physical health; any stigma experienced; how they had been coping with the illness; and what kind of support they needed and from whom. Interviews took place over a period of 2 months (August and September 2012). Sampling was discontinued after nine in-depth individual interviews (IDI), due to information saturation. Three focus group discussions (FGDs) were conducted using a structured topic guide to triangulate and further explore the findings from the in-depth interviews. The FGDs lasted approximately 1 hour and had six to nine participants. The focus group topic guide explored similar themes to those in the in-depth interviews.

All participants completed the self-administered SSQ 14 after the interview. The SSQ 14 was developed and validated as the first indigenous measure of CMDs in the Shona language in Zimbabwe by Patel *et al*. (1997). The specificity and sensitivity occurred at a cut-off point of 7/8. The 14-item SSQ has a high level of internal consistency (Cronbach’s alpha = 0.85). Each positive response is given a score of 1, with a maximum score of up to 14. The cut-off point for caseness is a score of 7 or above, and those scoring below are considered to be at low risk for developing CMDs. Individuals who score > 8 are considered at risk of CMDs; those who score 11 or above are at risk of severe CMDs. The total score correlates strongly with patients’ self-assessment of the emotional nature of their illness.

### Data management and analysis

Interviews were audio recorded and transcribed verbatim in Shona and then translated into English. Transcripts were entered into NVivo 8 (QSR International, Melbourne Australia), a qualitative data storage and retrieval program to facilitate analysis. Data were analysed using a thematic approach (familiarisation, identifying a thematic framework, indexing, charting, mapping and interpretation) (Smith & Firth 2011). A constant comparative method was used to identify the domains and subthemes emerging from the transcripts. Quantitative data obtained from the SSQ was captured using Epi Info 7 and analysed using Statistical Package for Social Sciences (SPSS), version 16. The Pearson correlation test for association was conducted at a significance level of α = 0.05. Simple descriptive statistics were conducted using SPSS.

## Ethical considerations

Ethical approval was obtained from the Joint Research Ethics Committee at the University Of Zimbabwe College Of Health Sciences (ref: JREC/156/12), Medical Research Council of Zimbabwe (ref: MRCZ/B/385/12) and the University of Cape Town Faculty Of Health Sciences Human Research Ethics Committee (Ref: 262/2012). Written permission was obtained from the matron in charge of the psychiatry unit. A private room was used for the interviews to ensure privacy and confidentiality. Participants were allocated unique identifiers, which were used instead of names in order to maintain confidentiality. Interview notes, consent forms and the audio recorder were kept in a locked cupboard for the entire study period to maintain confidentiality. Data were stored on a password-protected computer.

## Results

**FIGURE 1:**
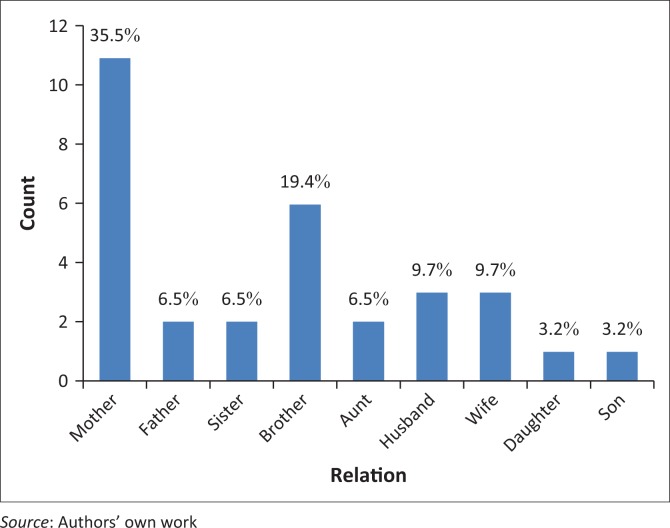
Relationship of caregiver to the patient.

Thirty-one family members (9 in the in-depth interviews and 22 in the FGDs) participated in the study. The mean age of the respondents was 44 years (range 22–69 years). Twenty-one (68%) were female and of these 11 (35.5%) were mothers to the patients, as shown in Figure 1 below. Eighteen participants (68%) lived in townships whilst 13 (42%) lived in low-density suburbs. The socio-demographic characteristics of participants are shown in Table 1.

**TABLE 1:** Socio-demographic characteristics of the respondents.

Variable	Frequency (*n*)	%
**Gender**		
Male	10	32.3
Female	21	67.7
**Marital status**		
Single	2	6.5
Married	20	64.5
Divorced	5	16.1
Widowed	3	9.7
**Educational status**		
Primary level	7	22.6
Junior certificate	4	12.9
Ordinary level	9	29.0
Advanced level	7	22.6
Degree level	4	12.9
**Place of residence**		
Low-density suburb	13	41.9
Township	18	58.1
**Religion**		
Catholic	8	25.8
Pentecostal	18	58.1
Traditional	1	3.2
Apostolic	2	6.5
Seventh-Day Adventist	1	3.2
None	1	3.2
**Employment status**		
Employed	8	26
Unemployed	23	74

*Source*: Authors’ own work

### Perceived impact of mental illness, coping strategies and needs

Six broad themes characterised the impact of mental illness on the family members. These were physical harm/illness, psychological and emotional impact, financial burden, material burden, social factors and stigma. The coping strategies used were seeking spiritual assistance, from the church and traditional healers; confrontation; resignation; and alcohol use.

### Physical harm/illness

Participants reported caregiving to be a challenging experience, as some of their family members living with mental illness became both physically and verbally aggressive to their caregivers, particularly when psychotic. As one participant stated:

‘Yes, it has affected us especially my husband he was assaulted and got injured. For me I was once assaulted by him but there is nothing to show but as for my husband he was really hurt.’ (IDI, mother, age 45)

Another participant stated:

‘There was a time when he stopped his medication and got very ill, to the extent that he was very aggressive and would lift up hoes and axes wanting to murder someone.’ (FDG, mother, age 66)

Less direct physical harm occurred to caregivers as a result of the stress they lived under. Caregivers described physical symptoms that they attributed to living with the daily burden of caring for the patient. One participant reported:

‘But now I realise that since I have been back here from the UK, I have lost a lot of weight, I can’t even wear my trousers, they are huge, they are now big, I just have lost so much weight because of the illness of my son and at times I can’t even eat it’s very difficult.’ (IDI, mother, age 55)

Participants reported failing to cope with the demands of the caring process and becoming stressed to the extent that they developed physical illnesses that they attributed to the patient’s illness. As one participant said:

‘I felt as if my hearing was impaired by the constant noise and today my back is painful – I have problems standing up in the mornings. My legs and my body – it’s like they have been dismantled, and I am always alone when my son relapses and starts shouting and beating everyone.’ (FDG, mother, age 54)

Another participant also explained how she had developed high blood pressure.

‘I was really affected a lot, because when we were staying with him he was always threatening that he would murder someone, several times the whole night. This affected me and I started having high blood pressure.’ (FDG, mother, age 61)

### Psychological/emotional impact

The psychological impact evident among the participants was the frequently used Shona idiom of distress: *kufungisisa*. This Shona term is translated as ‘thinking too much’ and is frequently associated with depression (Patel *et al*. 1997). As the following participant said:

‘Now I am always thinking too much and thinking for how long I will carry on looking after an adult. This is really eating me inside; you can’t be happy as a family when someone is not feeling well within the family.’ (IDI, mother, age 60)

One participant mentioned that she had noticed her child not meeting the same milestones as her child’s peers:

‘I can’t stop thinking too much, like you were given a child by God and you see him grow nicely and all of a sudden the child changes; especially when I see his peers, I see that my child would be doing this and that, but now he can’t – it’s really painful … ’[*starts crying*] (FDG, mother, age 45)

### Financial/material impact

Financial burden was expressed by many caregivers. It took several forms. For some, financial burden was experienced as a result of the caregivers having to leave their jobs in order to take on a caregiving role.

‘Me, I used to do things on my own to get money and never used to stay at home. I would go out to my aunt, who has a tailoring business, but I left everything to come here and stand by my son’s side.’ (IDI, mother, age 48)

Caregivers stated that, although treatment is free at government psychiatry hospitals, some had to buy medication for the patient, as it was frequently unavailable at the hospital.

‘Money is a problem, but you struggle as a parent to buy medications – but it’s hard and the father says we should spend even if we don’t have if it is going to benefit our child’. (IDI, mother, age 45)

### Social factors

Participants reported rejection by relatives because of their family member’s illness. A mother described how she was rejected by her mother and her own siblings due to her son’s illness and related disruptive behaviour. Some participants reported being unable to attend social gatherings because they had no one to leave the patient with, as relatives were not willing to assist with the care of the patient.

### Stigma

Caregivers reported being ostracised and isolated. They expressed that there was a lot of stigma associated with mental illness, which they attributed to lack of knowledge on the part of their relatives and the community at large. People in their communities believed that if you had mental illness you were a sinner. Women reported being blamed for the illness and being accused of bringing the illness into the home. One of the participants said:

‘From the time it started, my husband said: “This is from your family because in our family we have never encountered such things, and it’s you who brought it to the family.”’ (FDG, mother, age 55)

### Coping strategies

The most commonly used coping strategy was seeking spiritual assistance from both traditional healers and faith healers. This was frequently informed by the belief that the problem was linked to witchcraft. This was done to manage the patients’ symptoms and to allay the caregivers’ anxieties about the causes and progression of the illness.

‘It’s true we looked for help from prophets and ngangas [*traditional healers*], but it was her father who did that when he was still alive.’ (IDI, mother, age 63)

Some participants reported using confrontation to cope, whereby they shouted at the patient. As one participant said:

‘I shouted at him twice to say shut up, and he says, “do you want to hit me?” And I was really fed up with his behaviour and useless talk.’ (IDI, brother, age 36)

In other instances, the carers would ignore or avoid the patient. As one participant said:

‘Yes, I avoid the patient. I just go outside, because he doesn’t show respect to anyone, and I have come to a stage where I say I can’t live with him anymore – there is no peace. I am sorry, I cannot live with him anymore.’ (IDI, mother, age 56)

### Caregiver needs

Caregivers required financial assistance either from the government or donors (well-wishers).

#### Support groups

Some strongly felt that support groups for patients and caregivers would help them cope better. They went on to explain how support groups had been successful for people living with HIV/AIDS and felt that if they were to do the same for the mentally ill it would help. As one participant said:

‘If they do support groups like the one for people living with AIDS, it will be fine. Those people with HIV support each other, so we can also support each other.’ (FDG, mother, age 61)

#### Training

Caregivers said they required training to deal with psychological problems. They expressed a lack of knowledge about the condition their relatives were suffering from, because it was never explained to them. They wanted written materials so that they would read and have knowledge of the signs and symptoms of the condition. One participant said:

‘Written information is also helpful. There is someone who once gave me a book about this illness, and it helped me a lot; [*now*] we know that if [*things*] go this way, we do it this way.’ (FGD, brother, age 36)

Participants wanted doctors and nurses to have more time with them to explain the condition and explore the challenges they were facing in caring for their ill relative. As one participant said:

‘I think the time that we get with doctors and nurses is very limited, in such a way that you won’t understand what is going on. If you get into a session for 5 minutes, it becomes difficult to ask questions, and you are not told how you have to stay with the patient. I think that is a major challenge. I wish we could be given more time to be helped as carers and on how you are going to cope.’ (FDG, sister, age 22)

#### Provision of hope

Participants also wished to be given hope by the health professionals. They said that having someone talk to them and reassure them can allay their anxieties.

‘We want to be given hope just to say things will be better, things will be fine, don’t worry too much there are people with such conditions that have gone back to work, hope is important.’ (IDI, mother, aged 49)

Of note, the majority of caregivers said that this was the first time that they had been asked about how caring for their relatives was affecting them. As one participant said:

‘I have never been asked about how I feel about caring for my child, this is the first time and I wish there could be more people doing this.’ (IDI, mother, aged 58)

#### Shona symptom questionnaire results

All 31 participants (100%) completed all 14 items of the SSQ, as shown in Table 2. Twenty-nine (93.5%) reported having times at which they were thinking deeply or thinking about many things within the past week. Three (9.7%) of the respondents had suicidal ideation and a score of between 10 and 12 for CMDs. Two were patients’ mothers, 53 and 54 years old, whilst the third was a sister aged 33 years.

**TABLE 2:** Shona Symptom Questionnaire (SSQ) results.

Item	SSQ items (‘In the past week…’)	Yes *n* (%)	No *n* (%)
1.	Did you have times in which you were thinking deeply or thinking about many things?	29 (93.5)	2 (6.5)
2.	Did you find yourself sometimes failing to concentrate?	21 (67.7)	10 (32.3)
3.	Did you lose your temper or get annoyed over trivial matters?	21 (67.7)	10 (32.3)
4.	Did you have nightmares or bad dreams?	9 (29.0)	22 (71.0)
5.	Did you sometimes see or hear things that others could not hear?	8 (25.8)	23 (74.2)
6.	Was your stomach aching?	19 (61.3)	12 (38.7)
7.	Were you frightened by trivial things?	13 (41.9)	18 (58.1)
8.	Did you sometimes fail to sleep or lose sleep?	20 (64.5)	11 (35.5)
9.	Were there moments when you felt life was so tough that you cried or wanted to cry?	25 (80.6)	6 (19.4)
10.	Did you feel run-down or tired?	18 (58.1)	13 (41.9)
11.	Did you at times feel like committing suicide?	3 (9.7)	28 (90.3)
12.	Were you feeling unhappy with things you were doing each day?	19 (61.3)	12 (38.7)
13.	Was your work lagging behind?	24 (77.4)	7(22.6)
14.	Did you feel you had problems in deciding what to do?	24 (77.6)	7 (22.6)

*Source*: Authors’ own work

Ten participants (32.3%) were at a low risk of CMD, whilst 11 (35.4%) were at moderate risk and 10 (32.3%), including three with suicidal ideation, were at high risk of severe CMD, giving a total of 21 (67.7%) participants being at risk of CMDs as shown in Table 3. The participants at moderate and high risk were referred to the psychiatrist for further assessment and management. There was a significant positive association between female gender and being at risk of CMDs (*p* = 0.023).

**TABLE 3:** Shona Symptom Questionnaire (SSQ) total CMD scores for participants (*n* = 31).

CMD risk category	SSQ score ranges	*n* (%)
Low risk	3–7	10 (32.3)
Moderate risk	8–10	11 (35.4)
High risk	11–13	10 (32.3)

*Source*: Authors’ own work

CMD, common mental disorder.

## Discussion

### Outline of the results

This study highlighted the impact, coping strategies and needs of individuals caring for family members with bipolar affective disorder and schizophrenia in Harare, Zimbabwe. Despite having interviewed caregivers for both schizophrenia and bipolar affective disorder, data suggested that overall the impact, coping and needs of the caregivers of these patients were similar.

The majority (68%) of the participants were female and of these 35.5% were mothers. This supports the findings of Mhaule and Ntswane-Lebang (2009) in Mpumalanga Province of South Africa and Nasser-Hassan *et al*. (2011) in Egypt, who found that most caregivers (67% and 75%, respectively) were female. The role of women as caregivers is deeply rooted in cultural gender roles and traditional concepts of family life (DiGiloramo & Salgadode de Snyder 2008). Women’s caregiving is accepted as the norm in many cultures (Chitayat 2009), although the caring process can be a challenging experience, as patients can be physically and verbally aggressive to their caregivers.

Caregivers in this study frequently used the Shona term *kufungisisa* (‘thinking too much’) to describe the emotional or psychological impact of living with a relative with mental disorder. Thinking too much (*kufungisisa*) was also reported by participants in a study by Abas &Broadhead (1997) in their Zimbabwean study on depression. Numerous studies in HICs have shown that family caregiving causes stress and depression (Awad & Voruganti 2008; Wancata *et al*. 2006). Management of psychological distress among caregivers requires a multidisciplinary approach and consideration of the cultural context of the caregiver as well as the family member. There is an urgent need to come up with supportive interventions in the form of educating the family on the condition as well as exploring the potential impact of mental disorders on caregivers in resource-limited settings, a recommendation that was made by Shibre *et al*. in 2003. Group psychoeducation sessions could be helpful for maximising limited time and human resources. However, the limitations of family education need to be acknowledged; the findings from a study by Sefasi *et al*. (2008) in Malawi revealed that knowledge about the condition did not reduce caregiver burden.

Caregivers described financial burdens, which had a variety of forms and effects. This supports a finding by Shibre (2003) in Ethiopia that revealed that 74.4% of caregivers suffered financial burden and female relatives were more affected than males. Eighteen (68%) of the participants lived in townships, which are associated with low-income earners in Zimbabwe (Nkomo 2003). The findings on financial burden indicate the way in which carers frequently forego economic opportunities in order to take on caregiving roles, a pattern that can further entrench the poverty of a given household. Following the implementation of a community mental health programme by Basic Needs in rural Kenya, caregivers were able to take on income-generating roles, which were associated with significant increases in mean household income (Lund *et al*. 2013). This model could similarly be introduced in Zimbabwe to ease the financial burden on caregivers.

Caregivers reported being socially ostracised and reported a great deal of stigma associated with mental illness, which they attributed to lack of knowledge among their relatives and the wider community. This supports the findings by Shibre *et al*. (2003) in Ethiopia, who found that stigma associated with mental illness results in strained relationships within the families themselves, as well as difficult relationships with neighbours and the society at large. This problem has been attributed to the misunderstanding of mental illness aetiology (Shibre *et al*. 2003). Stigma has been found to be linked with depressive symptoms among the families of patients with mental illness (Lauber & Rossler 2007; Perlick *et al*. 2001), which is further supported by the findings of this study. There is a need to include other household members and community members more widely in health education about the causes of mental illness in order to reduce the stigma, blame and rejection that caregivers (and people suffering from mental illness) face in the community.

The caregivers who participated in this study used both emotion-focused and problem-focused coping strategies. This supports the findings of Ganguly, Chadda and Singh (2010) in their qualitative study, the results of which revealed that coping strategies were the same in caregivers of bipolar disorder and schizophrenia. The caregivers’ most commonly used coping strategy was seeking spiritual assistance from traditional healers and faith healers, because they believed that their relative’s mental disorder was linked to witchcraft. This supports the findings of the study by Shibre *et al*. (2001) on the perceived causes of severe mental illness and preferred interventions in Ethiopia, which revealed that mental illness was believed to be caused by witchcraft and that prayer was one of the interventions commonly used to cope with the conditions. Although the lay public and some caregivers of people with mental illness in Zimbabwe believe in witchcraft and supernatural powers as a cause of mental illness, biomedical models are now being considered. Medical management is now believed by many to complement the indigenous healing methods (Saravanan *et al*. 2008), as shown in this study.

Caregivers stated that support groups for both patients and caregivers could help them cope better. Support groups have been found to provide an opportunity for caregivers to share their experiences with people who are facing the same challenges (Mittelman *et al*. 2006). Another helpful ingredient mentioned by study participants is the provision of hope. In recognising and embracing the value of hope in coping with adverse situations and managing the uncertainty inherent in many chronic illnesses, the nursing profession most notably have been charged with the responsibility to inspire and engender hope in patients (Jevne & Nekolaichuk 2003). Mental health practitioners could be trained to impart hope in caregivers, as well as teaching caregivers appropriate coping skills as routine care. This will require practitioners to spend more time with both patients and caregivers. The findings of Jonsson *et al*. (2010 in Canada revealed that hope was crucial for family members living with a family member with mental illness, a finding also supported by Bland and Darlington (2002).

Of particular note is that the majority of caregivers in this study expressed that this was the first time they had been asked about how caring for their relatives was affecting them. This finding is likely an indication of their sense of isolation and lack of support. This may also indicate competition between the caregivers and the patients for care and attention from the healthcare professionals.

The findings of this study point to a number of recommendations. Firstly, healthcare workers need to pay more attention to the needs of caregivers by providing them with information and counselling as routine care. This recommendation was also made by Shibre *et al*. (2003) in their cross-sectional study on caregiver burden in Ethiopia. Caregivers of patients with both bipolar affective disorder and schizophrenia have been found to require emotional support and psychoeducation for them to effectively provide care and support to their mentally ill relatives (Jonsson *et al*. 2010; Mangliano *et al*. 2000 Mehra *et al*. 2005). Psychoeducation has been shown to be effective for family support in schizophrenia in many other settings and could also be adapted to be culturally sensitive in Zimbabwe, as well as potentially other countries in sub-Saharan Africa.

A second broad recommendation pertains to broader health system changes. There is a critical shortage of mental health professionals in Zimbabwe. Task shifting may potentially be effective in alleviating this critical shortage of human resources in mental health (Fulton *et al*. 2011). Lay community workers have the potential to provide psychosocial and psychological interventions as part of the primary and secondary prevention of Mental, Neurological and Substance use (MNS) disorders in LMICs (Mutamba *et al*. 2013), and they can be used to support patients and caregivers in the community through identification of signs of relapse and providing psychoeducation, among other things. Adequate training and supervision of these non-specialised cadres by specialised cadres is essential for them to effectively support mental health services. Family caregivers can also be involved in detection of mental health disorders and to encourage their relatives to go for treatment early. This has been done successfully in India as well as in Chile and has been shown to reduce caregiver burden (Srinivasa *et al*. 2005).

Th**e** strength of this study is that it presents new knowledge on the experiences of family caregivers of patients with mental disorders who are attended to in psychiatry hospitals and their coping strategies and needs in Zimbabwe. This area has received very little prior research attention. Furthermore, by combining qualitative and quantitative methods we were able to triangulate the data and validate our findings: the subjective qualitative reports regarding caregiver burden were clearly evident in the level of psychological distress measured on the SSQ.

There are a number of limitations to our study. Firstly, the sample size was small and participants were purposefully selected; therefore their views may not reflect the views of all the caregivers of patients with mental disorders in Zimbabwe. Secondly, we did not collect information on the ages of the family members living with a mental illness. Thirdly, we did not enquire about violent behaviour by caregivers towards their mentally ill relatives. Fourthly, the study population was also that of a minority who had access to a psychiatry referral hospital, which might not reflect the challenges and needs of those who have no access to psychiatry services. The participants were the relatives of patients attending a referral centre, who might therefore have more severe illnesses, thereby increasing the caregiver burden. Compared to the catchment area of the hospital, the number of beds and outpatients services is grossly inadequate for the needs of the population it serves. This might reflect that there could have been a number of patients in the community who were not able to access services in this hospital, which might increase the likelihood of their caregivers experiencing more burden. Fifthly, the study was done in an urban setting, thereby affecting generalisability to rural settings. In light of these limitations, it is therefore recommended that future studies address these limitations and, among other approaches, quantitatively measure burden in more representative settings.

Despite the limitations, our findings indicate that it is critical to address the needs of caregivers in order to help them provide adequate support to their family members and prevent the occurrence of CMDs. The findings have generated hypotheses for more research to quantify the impact of mental disorders on caregivers across Zimbabwe, including those who are not able to get access to psychiatric hospitals. Intervention studies with robust designs, such as randomised controlled trials, need to be conducted to assess the effectiveness of psychoeducation and other supportive interventions for caregivers in the care of family members with mental disorders in Zimbabwe. Task shifting through use of lay workers has been found to be potentially effective in the provision of mental health services in other countries and therefore may be effective as an intervention to support caregivers in the community in Zimbabwe.

## Conclusion

Caregivers of patients attending psychiatry hospitals in Zimbabwe require support from healthcare professionals to help them cope with caring for their family members with a mental disorder. These findings are useful for policy formulation to redesign or resuscitate appropriate mental health intervention strategies for caregivers of patients with mental disorders in Zimbabwe.
